# The constant conflicts of opioid prescribing – GPs’ views on treating chronic pain with opioids in primary care: a reflexive qualitative analysis

**DOI:** 10.1080/02813432.2026.2665697

**Published:** 2026-05-05

**Authors:** Anna Svensson, Hanna Ljungvall, Thomas Kempen, Sofia Kälvemark-Sporrong, Magnus Peterson

**Affiliations:** a Academic Primary Health Care, Region Uppsala, Uppsala, Sweden; b Department of Public Health and Caring Sciences, Section of Family Medicine, Uppsala University, Uppsala, Sweden; c Department of Social Work, Uppsala University, Uppsala, Sweden; d Utrecht Institute for Pharmaceutical Sciences, Utrecht University, Utrecht, The Netherlands; e Department of Pharmacy, Social Pharmacy, Uppsala University, Uppsala, Sweden

**Keywords:** Pain, general practitioner, ethical dilemmas, primary care, opioids

## Abstract

**Background:**

Despite limited evidence supporting the long-term effectiveness of opioid therapy and existing recommendations on multi-disciplinary treatment, patients with chronic non-cancer pain (CNCP) continue to receive opioids. In Sweden, most prescription renewals for CNCP are issued by general practitioners (GPs).

**Aim:**

To explore GPs’ experiences with and understanding of opioid prescribing for CNCP and their views on multi-disciplinary collaboration.

**Methods:**

Semi-structured interviews were conducted with GPs and one GP-trainee in Region Uppsala, Sweden (February–June 2022). Topics included long-term opioid treatment (LTOT), opioid tapering, prescription renewal and multi-disciplinary collaboration. The interviews were recorded, transcribed and thematically analyzed.

**Results:**

Twelve participants including one GP trainee with a mean experience of 13 years were involved. Two main themes were developed. (1) *To endure the ethical conflicts of opioid prescribing practices*, where the GPs negotiated ethical conflicts arising in the intersection between patient needs, personal values and clinical guidelines regarding LTOT. (2) *The last resort*, where the GP’s role was conceptualized through the lens of systemic healthcare structures, perceived patient demands, expectations from fellow healthcare providers and the internal expectations held by themselves.

**Conclusions:**

GPs experienced moral distress prescribing opioids and reinforced stigma toward patients with CNCP and LTOT. These dynamics hinder treatment reassessment and biopsychosocial evidence-based care. Perceived expectations of GPs as the ‘last resort’ may perpetuate opioid use and impede collaborative care with the patient and multi-disciplinary teams.

## KEY POINTS

- Opioid prescribing involves significant ethical dilemmas for general practitioners (GPs).
- General practitioners face challenges balancing opioid risks with patient expectations and work load.
- Tapering long-term opioid therapy (LTOT) is regarded as a time-intensive process and is often deprioritized.
- The identity of the GP as a comprehensive healer is challenged within the context of chronic non-cancer pain (CNCP) and opioid prescribing.
- General practitioners infrequently utilize multi-disciplinary approaches for the care of patients with CNCP and LTOT.

## 1. Introduction

Chronic pain, defined as pain persisting for more than three months [1], represents a major contributor to the global burden of disease, affecting approximately 20% of the adult population [2,3]. It ranks among the top five causes of years lived with disability and imposes a substantial socioeconomic burden on health care systems worldwide [3]. Owing to its multifaceted nature, an individualized biopsychosocial approach to assessment and treatment of chronic pain is recommended, within which pharmacological interventions may serve a supportive role [4–7]. Evidence for sustained analgesic benefit from long-term opioid therapy (LTOT) in patients with chronic non-cancer pain (CNCP) remains weak. Opioids are generally recommended only for acute or cancer-related pain [8,9]. Hence, opioid use beyond three months for CNCP is increasingly questioned [5,8, 10–12]. Increased risks of morbidity and mortality including prescription opioid use disorder and overdose are important issues [6,13]. Despite these risks, patients with CNCP continue to receive LTOT [10,14,15].

From the mid-1990s, rates of pharmaceutical opioid prescribing increased in many Western countries due to the perception that opioids were safe and non-addictive [16,17]. This trend was further reinforced by aggressive marketing and clinicians’ perceived obligation to treat pain [16,17]. In Sweden, although the level of opioid prescribing has stabilized since 2016, a notable shift has occurred from weaker opioids, e.g. tramadol, toward stronger agents, e.g. oxycodone [18–20]. Concurrently, an increase in oxycodone‑related mortality has been observed, corresponding to approximately one-third of the proportion reported in the United States [21].

Contemporary scientific priorities center on strategies to prevent inappropriate prescribing and implement evidence-based tapering protocols [6,7,22–27]. Currently, available tools for determining which patients benefit from LTOT in CNCP are incomplete, complex and place considerable responsibility on the physician [6,28,29]. A recent systematic review of general practitioners’ (GPs’) attitudes toward opioids for chronic pain underscores the challenges of managing CNCP with opioids in primary care [28]. Contributing factors include the perceived obligation to relieve pain, the responsibility for society at large, and the need to weigh analgesic efficacy against potential adverse effects. GP-related factors further complicate decision-making. These include prior experience with opioid tapering, conflicts with patients regarding opioids and feelings of powerlessness [28]. This sense of powerlessness encompasses experiences of opioid prescribing. Negative experiences include the sense of patients being ‘dumped on GPs’, a ‘lack of alternatives’ including non-pharmacological options as well as ‘lack of knowledge and evidence/education’, ‘lack of legislation and appropriate protocols and contracts’ and ‘lack of time’ [28].

Moreover, evidence guiding the implementation of opioid tapering remains limited. However, existing recommendations emphasize shared decision-making, individualized and closely monitored tapering strategies, and multi-disciplinary collaboration [30–34]. Key challenges include communication barriers and insufficient healthcare resources to support safe and structured tapering [33,35].

Chronic non-cancer pain is predominantly managed within primary care where pain as a symptom constitutes one of the most common reasons for patient consultations in Sweden [36–38]. Prescribers in the Swedish primary care setting, that is GPs, GP trainees and locum doctors, account for the majority of opioid prescription renewals following the initial prescription [18].

This occurs within a healthcare system that is, at least in principle, designed to promote multi-disciplinary collaboration and support the application of a biopsychosocial approach in primary care. This is facilitated by the presence of physiotherapists and psychologists in most primary care centers (PCCs) [39]. Nevertheless, previous government-funded initiatives to implement such collaboration for chronic pain management have failed [40]. Persistent challenges include, among others, inadequate coordination of care for chronic conditions and a severe shortage of GPs, estimated at 40% below required levels in 2022 [39]. Consequently, the responsibility for establishing multi-disciplinary collaboration rests with individual PCCs, resulting in highly variable implementation and outcomes. Primary care in Sweden is publicly funded, regulated by the authority of regional healthcare governance and the patient pays a small fee (approximately 20 euro) for health care visits, regardless of whether it is provided by public and private actors.

Three studies [41–43] have examined how Swedish GPs experience and justify opioid prescribing practices for CNCP, despite not being first-line treatment according to recommendations [5]. Bendtsen et al. identified two main categories of dilemmas: concerns about abuse and addiction, and uncertainty regarding drug appropriateness [43]. Ekelin and Hansson reported that renewing weak opioids without prior consultation often triggered ethical conflicts and emotional strain toward patients, colleagues and physicians themselves [42]. Ljungvall et al. further highlighted the complex transition from initial prescribing to long-term use shaped by opioid dependence and withdrawal-driven pain. Other physician and patient factors and organizational barriers to person-centered care [41] are also implicated. Collectively, these findings underscore the need for structured support and enhanced education in pain and addiction medicine.

Hence, there is a need to explore GPs’ experiences with, and attitudes to, prescribing opioids for CNCP and multi-disciplinary collaboration in a Swedish primary care setting.

## 2. Aim

We sought to explore Swedish GPs’ experiences with and understanding of opioid prescribing for CNCP and their views on multi-disciplinary collaboration for the management of CNCP and LTOT.

## 3. Materials and methods

### 3.1. Design and theoretical framework

Individual interviews were conducted using a semi-structured interview guide to collect qualitative data. The consolidated criteria for reporting qualitative research (COREQ) checklist was used in the reporting of the study [44].

As an ontological and epistemological foundation, we adopted reflexive naturalism [45,46]. This treats the natural world as real and knowable via science (*naturalism*) while acknowledging how knowledge is produced by situated researchers (*reflexivity*). This approach enabled identification of themes grounded in empirical data while recognizing the constructive nature of interview data as well as the role of researchers’ preconceptions in shaping interpretation [45].

### 3.2. Analytical concept

To deepen the analytic process and strengthen theoretical transferability, we applied an analytical concept grounded in professional bioethics as articulated by Beauchamp and Childress [47]. Rooted in common morality, i.e. the set of universal norms applicable to all persons across all contexts, there are four interrelated clusters of moral principles that can serve as a guide for clinical practice. These are

1. respect for autonomy which requires the recognition and support of decisions that are intentional, informed, and made without undue external or internal influence,
2. non-maleficence, i.e. an obligation to avoid causing harm or imposing risks, but does not imply a duty to actively provide benefits such as health care,
3. beneficence, i.e. a foundational ethical principle in health care, encompasses the obligation to promote benefits, prevent harm and balance benefits, risks and costs to achieve optimal outcomes,
4. justice, i.e. ‘a cluster of norms for fairly distribution benefits, risks, and costs’ which is closely tied to health care financing and delivery [47].

### 3.3. Setting, participants and recruitment

The study was conducted in Region Uppsala, located approximately 60 km north of Stockholm. Region Uppsala is comprised of 410,000 inhabitants and more than 50 primary health care centers which are responsible for ensuring access to and quality of care. At the time of the study, approximately, 170 GPs were employed within Region Uppsala [48]. The participants were invited to participate via email. Purposive sampling was used to recruit GPs or GP trainees. The intention was for heterogeneity with variation in working experience, type of employment (permanent, temporary), size and geographical location of health center (urban/rural). Similar to the majority of PCCs in Sweden, all participants had access to multi-disciplinary collaboration with physiotherapists and psychologists/counselors within their own PCC. During the recruitment process, assistance was obtained from an experienced pain specialist who was accustomed to assessing referrals from primary care and had extensive knowledge of GPs in the region. During the interviews, the informant was asked for suggestions on suitable individuals to interview, for example: ‘Do you know anyone who holds a different view on opioid prescribing in primary care?’. Furthermore, a primary care manager was consulted regarding a suitable informant from that specific PCC. The interviews were conducted in Swedish. In line with Clarke and Braun’s reflexive approach, our aim was not to achieve saturation in the sense of exhausting all possible themes, but to ensure sufficient depth and richness to address the research question. After iterative coding and theme development, additional data were not generating substantially new conceptual insights, indicating that our dataset was adequate for a meaningful and nuanced analysis [49].

### 3.4. Data collection

Interviews were conducted face-to-face, by telephone or via online meetings by AS using a semi-structured interview guide (Appendix 1). The guide was developed by the research team based on the study aim, the clinical experience of AS, MP, TK and HL, and previous literature [28,41,42]. To capture diverse experiences, the guide was built around broad open-ended questions such as ‘What is your view on opioid prescribing for chronic pain in primary care?’. The interview guide was then supplemented by probing questions to elicit deeper responses. All interviews were audio-recorded and transcribed verbatim by AS. The quotations were translated by AS with support from at least one additional co-author and were subsequently language-edited by both Microsoft M365 Copilot (a GPT‑5-based model) and a native English speaker. All final phrasings were reviewed and approved by the authors. The transcriptions were pseudonymized to ensure confidentiality.

### 3.5. Authors’ positionality

AS is a PhD candidate and an experienced GP with post-graduate training in pain medicine. HL is a clinical social worker and researcher within the field of pain and substance use disorders with extensive clinical experience in opioid use disorder. SKS is a professor of social pharmacy with expertise in medication prescribing from a societal perspective. TK is a clinical pharmacist with experience in opioid tapering within primary care and holds a PhD in enhanced medication safety. MP is a senior lecturer, GP and pain specialist. The authors’ experience in qualitative research varies: none (AS), some (MP, TK) and extensive (HL, SKS).

### 3.6. Data analysis

**Figure 1.**
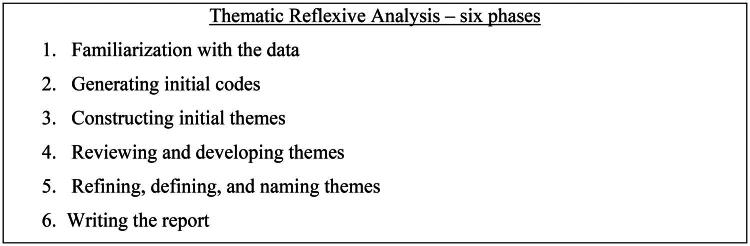
The six analytic phases of thematic reflexive analysis [30].

The transcribed interviews were imported into NVivo 13, for coding and analysis. Data were analyzed in six phases according to Braun and Clarke as outlined in Figure 1 [46,50,51].

AS and HL engaged in repeated readings of all transcripts supporting a deep understanding of the data, noting initial impressions and underlying meanings. These early impressions and notes were discussed on several occasions. Based on this process, AS conducted inductive coding, generating both semantic codes – capturing explicit meaning – and latent codes – reflecting underlying interpretations. The coding was reviewed and discussed with HL and SKS who read three transcripts selected for their variation, contributing further insights. Preliminary themes and subthemes were generated from the codes. This process involved AS, HL, SKS and MP, who had read all transcripts. To ensure transparency, AS maintained an audit trail consisting of meeting notes and manuscript versions from inception to finalization. Interpretation of data was discussed repeatedly within the group and was reflected on in relation to the researchers’ different preconceptions and the theoretical framework. This generated the final themes and subthemes. Consensus on themes was reached during meetings among AS, HL, MP and SKS. The third author, TK, reviewed the audit trail and consistency between codes and themes.

### 3.7. Ethical considerations

According to the Swedish Ethical Review Act (2003:460, 1–22§ [52]), ethical approval was not necessary for this study as the research neither affected the participants physically or psychologically, nor included the handling of sensitive personal data according to the GDPR [53]. The participants received both oral and written information about the study. Their potential participation was voluntary, confidentiality was guaranteed and they could decline to answer any questions or withdraw from the interview at any time. All provided written informed consent.

### 3.8. Trustworthiness

To enhance trustworthiness of the study, the authors engaged in reflexivity, i.e. how their own expectations on data influenced the research process and interpretations [54]. This included keeping an audit trail in which each step of the analysis and decisions regarding interpretation and abstraction were systematically documented. Furthermore, involving four researchers with diverse clinical backgrounds and research expertise facilitated peer debriefing and researcher triangulation throughout the analytical process [54]. Moreover, a semi-structured interview guide was used. This included follow-up summaries of the participants’ statements to clarify response ambiguities and probing questions to obtain more nuanced responses.

## 4. Results

In total, 15 GPs and residents were invited and 12 agreed to participate. Two declined to participate due to time constraints and one did not respond. The age of the participants ranged between 33 and 67 years old. Seven out of 12 participants were female (Table 1). The average length of work experience was 13 years (range 2–34 years), with one participant being a GP resident instead of a GP. The interviews were conducted between February and June 2022. They were mostly face-to-face in the participant’s professional setting (*n* = 8) instead of digitally or by phone (*n* = 4) and lasted on average 46 min (range 35–57 min).

Two main themes and five subthemes were identified:

1. *To endure the ethical conflicts of opioid prescribing practices* with three subthemes: (*4.1.1) The battlefield of opioid prescribing, (4.1.2) someone else*’*s decision – the responsibilities of opioid prescribing* and *(4.1.3) legitimatizing opioid prescribing practices.*
2. *The last resort* with two subthemes: (*4.2.1) Lonely…* and *(4.2.2) … and indispensable.*

### 4.1. To endure the ethical conflicts of opioid prescribing practices

The narratives about opioid therapy and opioid prescribing practices for CNCP were filled with ethical dilemmas which the GPs had to manage in a constant balancing act. They were often caught in the juxtaposition of organizational demands such as lack of time and resources for multi-disciplinary rehabilitation, and the need or wishes of the individual patient. The most salient conflict described was when the patient wanted to continue his/her opioid therapy although the GP was reluctant to prescribe. When this occurred, the GPs were confronted with a situation where they had to balance the principles of respect for autonomy, non-maleficence, beneficence and justice when making their decision on whether to continue prescribing the opioid treatment. The prescribers employed various strategies to achieve this and to endure the conflicts that emerged, which are further explored in the sub-themes below.

#### 4.1.1. The battlefield of opioid prescribing

This subtheme illustrates the experienced consequences of questioning the opioid treatment and potential conflicts with the principle of respect for autonomy and how these consequences could pose a barrier for tapering opioids.

All GPs described the experience of continuing to prescribe opioids despite unclear indications for continued prescribing and narratives about opioid tapering were uncommon. This could be understood as a way to avoid open conflicts with the patients given the sensitive topic of opioids and their association with opioid use disorder and dependence. Several GPs were concerned about patients on opioids being manipulative, unreliable and aggressive, making it hard to provide a just and correct assessment regarding the effects of the treatment. To prescribe or taper was in some cases experienced as being a battlefield. The GPs used metaphors such as ‘struggle’, ‘weapon’, ‘victory’ and ‘loss’ to depict the situations. For example, ‘I choose my battles’ (GP 4) or ‘they [the patients] often use pain as a weapon to get what they want’ (GP 9).

One way to balance whether or not to enter a potential conflict was to emphasize the importance of having a motivated patient before considering tapering opioids. The GPs narrated how they often strove to involve the patient and to consider their opinion and experiences when evaluating the effect of opioids and potential opioid tapering. Otherwise, many GPs expressed the view that there was no point trying to taper the opioid. This approach could be understood as a way to respect autonomy as well as engage in shared decision-making.

I try to probe them, where are they? [patient:]’well, I take it, I’m wearing it [buprenorphine patch], but I don’t know if it really helps’ and then I have a basis, if someone is wavering in their answer then I go for it, then I take the opportunity and talk about [to the patient:] ‘why not start without?’. But if I get an answer that it has a convincing effect [according to the patient], ‘I can’t live without that’/…/then I put it directly in a compartment in my head that ‘no, there is no point doing that’. (GP 4)

One strategy to avoid a potential conflict, even though the GP suspected a patient might have problematic opioid use, was to continue prescribing opioids. This was justified as a way to safeguard the therapeutic alliance and not cause maleficence by exposing the patient to severe withdrawal or pushing the patient to source illicit opioids for medications. Hence, non-maleficence was used as an argument. In these situations, several prescribers adopted a paternalistic approach, where they tried to be in control of the opioid prescription and the patient’s opioid use. This included informing the patients about the framework they applied for prescribing opioids and other addictive drugs but also threatening the patient that non-compliance would result in the termination of opioid treatment, as narrated by GP 3:

If you overuse and suddenly run out, then you are out. You won’t be able to call and say you need more/…/They really understand that they have to manage this because if they don’t follow the treatment plan, I will stop the medication/…/and it usually works well actually/…/they can’t just blame it on ‘my dog ate the tablets’ or ‘my mom took the tablets’ or ‘something happened to the tablets’ because no matter what happened, I won’t buy it. (GP 3)

However, when the GP decided to terminate the opioid treatment, this was perceived as entering a battlefield, challenging the patients’ wishes and overruling their autonomy, often justified by the addictive nature of opioids. This led the GPs to consider the patients not competent to make decisions regarding opioid therapy.

I’m prying and plucking and talking and finally I say ‘it doesn’t help, it’s not helpful with morphine in this condition because what has happened is an addiction’. (GP 10)

In such cases, the GPs described the need to take a determined and directive role in guiding the tapering process once the decision to stop prescribing had been made. This entailed a consciousness of the risk of jeopardizing the patient’s trust, as narrated by GP 10:

I have had many pain patients at the clinic and have had to realize that ‘this is how it is.’ It has not been without complications, and there have been many issues, including angry patients who deregister and want to go to someone else, feeling angry and offended, and all sorts of things. (GP 10)

#### 4.1.2. Someone else’s decision

This subtheme describes GPs’ understanding of opioids as harmful and illustrates how the ethical principle of non-maleficence may conflict with the ethical principle of justice.

Opioids were portrayed primarily as addictive and ineffective for treating CNCP considering the lack of evidence and the well-known risks with opioids which they often exemplified by the opioid epidemic in the U.S.A. There were concerns about contributing to iatrogenic opioid use disorder or overdosing, both intentional and unintentional. In addition, apprehensions were raised about patients committing suicide if the GP ended the prescription without providing adequate support. Thus, the potential for serious harm or maleficence arose both from prescribing and discontinuing prescribing, highlighting why ending a perceived inappropriate prescription did not always entail the actual termination of the opioid prescribing.

You can’t just stop/…/give people their opiates either, so they have to get it from somewhere, I mean, they may not get the prescription but they have to get an assessment quickly so they don’t just go cold turkey, there are big risks with that. (GP 12)

Moreover, the decision not to discontinue a treatment perceived as potentially harmful could, in some cases, be understood in light of the GPs’ perceived responsibility for the allocation of health care resources. This was an argument for deprioritizing tapering. Discussions about, and the process of, opioid tapering were described as arduous for both the patient and the GP and were therefore impeded by the GPs’ overwhelming workload. Consequently, the GPs feared that the process of motivating and conducting opioid tapering would compromise other patients’ needs as described below:

Patients can get a better pain status and functional state after a longer period of time because then it is about being able to be such a support, until it is better/…/and there we are probably lacking as health centers/…/it will be an increased job and more difficult for the patient for a period of time/…/and then there are displacement effects for other patients and the patient feels bad, you need to maintain closeness for the patient to cope. (GP 2)

To balance the non‑maleficence considerations inherent in opioid prescribing against the perceived injustice of allocating substantial resources to tapering, the GPs sought to minimize patients’ risk of harm by maintaining close oversight of opioid use and preventing overconsumption. They described implementing a range of monitoring strategies to manage opioid prescribing. Furthermore, the GPs often referred to prescription renewals as effectuating someone else’s decision, thereby disclaiming responsibility.

I find that most patients to whom I prescribe opioids are actually inherited patients/…/I think that there are not many new prescriptions that we initiate. I don’t think it’s just me; I think it’s generally the case. Most are prescription renewals that we try not to do routinely; instead, we try to assess the situation. We try to have routines here so that it doesn’t just become a prescription renewal among many others if it’s not a patient you know. However, it still happens sometimes. (GP 6)

All GPs considered common local guidelines beneficial in minimizing the overall opioid prescription rates, hence reducing the risk of opioid-related harms to the local population. Several GPs asked for national and regional guidelines that they could rely on when denying their patients continued opioid treatment. This could be interpreted on the one hand as a way to avoid engaging in conflict-filled discussions about opioids and on the other hand as a means of alleviating the discomfort associated with prescribing a medication perceived as risky and harmful. Thus, they were managing the ethical dilemma of potential maleficence without having to take full responsibility for denying the patient the wanted treatment.

#### 4.1.3. Legitimate opioid prescribing practices

This subtheme illustrates how continued opioid prescribing may be understood as an ethically justified decision.

Although all GPs considered opioids an ineffective and potentially harmful treatment for CNCP, they talked about experiences from situations where the prescription was dissociated from ethical conflicts. For example, in palliative care or in elderly people where there were few other treatment options. Though some GPs reflected over potential harmful use and cognitive side effects even in these situations, the patient’s right to pain relief outweighed the risks.

Prescribing opioids was also considered legitimate for patients with clinical and objective findings. These could include osteoarthritis awaiting surgery or patients where significant findings were found on MRI of the cervical spine or lumbar spine in a patient communicating severe pain.

I had a man/…/in his 40s, who had a calcified herniated disc visible on MRI, and it looked terrible. It was like, ‘you should be on morphine all year round,’ but he chose not to take it. He only uses acetaminophen, and he’s in a lot of pain, but he says, ‘no, I don’t want to deal with morphine medications.’ Then there are others who don’t have clear radiological findings but are very eager to get strong medications prescribed. The range is vast in how people choose to manage their own pain. (GP 1)

This quote illustrates how the GPs judge the patients’ pain and approaches to pain management. The patient’s pain becomes credible to the GP and the principle of non-maleficence is overshadowed by what the GP perceives as beneficence and opioids a potentially legitimate treatment.

Furthermore, when reflecting on the beneficence of prescribing a potentially harmful drug to younger patients, the GPs emphasized that all other treatment options must first be exhausted and that opioid use should be endorsed by a pain specialist. Even if GPs felt uncomfortable with the pain specialist’s judgment regarding opioid dosage, if the pain specialist had given his/her blessing, the prescription continued.

### 4.2. The last resort

This theme illustrates the GPs’ role within the health care system and in relation to the patient, interpreted through the experiences of prescribing opioids for CNCP.

GPs experienced their role as being the last resort in the healthcare system. This brought a sense of responsibility to care for all patients at the health center and to ensure access to compassionate, empathetic healthcare providers. This sense of responsibility meant taking full ownership of patients’ care; hence, delegating tasks to other professionals was considered difficult. GPs viewed themselves as best equipped to manage the complexities of CNCP and LTOT. However, regarding the treatment of chronic pain in general and LTOT in particular, the GPs considered primary care to be poorly equipped for this due to lack of resources and support. Opioid prescribing could also be a means to fulfill the GP’s obligations, i.e. being a compassionate care provider enacted as alleviating pain by using the prescription pad according to the patient’s wishes. In all, this generated feelings of being alone but also of being indispensable, as narrated in the subthemes below.

#### 4.2.1. Lonely…

This subtheme illustrates the feeling of being abandoned by colleagues, the system and the health care organization when trying to treat suffering patients without tools to provide care to replace the opioid treatment.

The GPs seemed to perceive a sense of isolation and loneliness in their role. CNCP was considered a complex condition, involving both affective symptoms as well as pain. Opioids were partly acknowledged to provide pain relief but mainly to alleviate affective suffering and withdrawal symptoms. This could explain the commonly described frustration of not being able to get support from, for example, pain and addiction specialists.

I wish the pain clinic wasn’t so picky with referrals. If I say that here is a patient who needs their help, they should trust that instead of saying, ‘no, you have to manage on your own.’ (GP 3)

A sense of loneliness was also described in cooperation with other health care providers within the primary care health center. Considering CNCP, the GPs said they strived to involve other professions such as physiotherapists and psychologists, acknowledging CNCP as a diagnosis where a biopsychosocial approach is essential.

Well, I find it a bit difficult to manage, partly because I don’t really feel like a specialist when it comes to explaining it to the patient. It’s hard to fit it into a regular appointment. But then you can at least refer the patient to a colleague who can have that discussion, and I think that’s incredibly valuable because you really need several people involved with these patients/…/To give them the best possible treatment and, if possible, to reduce the use of opioids a bit, if that’s the plan/…/A: Then it would be good if someone else is on board, not just you GP 6: Yes. A: And the patient. GP 6: Exactly, preferably a physiotherapist and a psychologist as well. (GP 6)

Despite this, some GPs were reluctant to engage in multi-disciplinary collaboration. This was partly attributed to previous experiences where physiotherapeutic interventions had failed to produce meaningful progress leading to patients being referred back to the GP. These referrals were often accompanied by expectations from both other healthcare professionals and the patients themselves for further pharmacological treatment, making it challenging to refrain from prescribing opioids. As described by GP 7:

But then they come back when it doesn’t work/…/and then it becomes my problem again, so to speak/…/The sad part is that I’m pressured into something I’m not really proficient in,/…/but at the same time, if you are to send them somewhere, it’s not certain they will be received. (GP 7)

Thus, seeing themselves as the last resort involved taking responsibility for questionable prescriptions and even initiating opioids in the absence of further referral pathways or viable therapeutic alternatives. In this sense of abandonment, and fearing criticism for the provided treatment, the GPs spoke about the necessity of meticulous record-keeping. By this, they carefully argued for why they continued such a prescribing.

you have to try to justify it in the medical record/…/so that colleagues can see that you have thoroughly reviewed and linked to your journal entry. (GP 8)

Despite considerable efforts to justify opioid prescriptions – efforts which included conducting what they perceived as adequate risk–benefit assessments in collaboration with the patient – the GPs expressed ambivalence regarding their decisions. Left alone with the responsibility of accommodating a decision that went against clinical guidelines in favor of providing what was perceived as an adequate treatment aligned with the patient’s wishes.

You don’t feel really good, you don’t feel really satisfied no matter what you do, it doesn’t feel really good to prescribe, because somehow I feel that it’s not right, but if I say no then it doesn’t feel really good either/…/so you like, you won’t be satisfied no matter what you do/…/. (GP8)

#### 4.2.2. …and indispensable

This subtheme illustrates how the GPs, while expressing abandonment, expressed proficiency in treating patients with complex diagnoses. This included balancing risk–benefit and having a holistic approach.

The GPs considered themselves to be more suited to prevent further opioid treatment than other professions, given their medical expertise and their knowledge of the patient’s history. As described in subtheme *Lonely…*, some GPs experienced that when other professions were involved, this could open up for expectations on renewed opioid treatment from both the patient and other health care providers. They expressed the view that the GP must assume a position of leadership and of being in charge of the medical treatment.

The prescribers described that it was hard to discuss both pain and opioids in a compassionate and non-judgmental way, an approach that they strived for and identified with. This could be understood within the role the GPs see as theirs, i.e. as someone who offers tangible interventions within the biomedical framework, often in the form of medications. To merely inform the patient or listen to the patient’s complaints was not considered satisfactory considering the perceived expectations on the GP to act and intervene.

Sometimes I just can’t avoid continuing to prescribe/…/And somewhere in all of this, I guess I’ve come to accept my own limitations—that I can’t offer them a better treatment because everything has already been tried. (GP3)

There was a narrative of the GP as the doctor who reassures, takes the patient seriously and creates security in a fragmentary health care system. It was emphasized that no one else in the health care system – from pain specialists to pharmacists or physiotherapists within their own health center – has the same holistic perspective on the patient. Others may also lack the ability to engage in discussions about opioid treatment. This reassurance was fostered through physical visits with the aim of achieving the patient’s trust. Trust was established by spending extra time with the patient, reviewing the medical history, and conducting a thorough examination of the patient’s health condition. This was considered a good breeding ground for an alliance that could support dose reduction and, ultimately, the discontinuation of prescribing. The relationship between the GP and patient was experienced as unique. In this, the GP could be perceived as indispensable when discussing the role of a team:

of course a team would help me/…/But/…/just as I say, patients give completely different histories to a nurse, completely different histories to a pharmacist/…/than to me, or I ask questions in a different way/…/I believe that I am experienced enough to get much more out of people than they want to tell (laughs)/…/but I am experienced, I think that now I can form an alliance and I can extract information that would never come out if it were a pharmacist calling up/…/But on the other hand, I know everything about that person/…/that he has had an amphetamine addiction before, and so on. Then I understand that this is something, it plays a role here. I shouldn’t mess with your Tramadol because I know so much more, knowledge and details through follow-up. (GP 4)

## 5. Discussion

In this study, we explored Swedish GPs’ experiences with and understanding of opioid prescribing practices including opioid tapering for patients with CNCP. The analysis identified two main themes: (1) *To endure the ethical conflicts of opioid prescribing practices* and (2) *The last resort.* The first theme illustrates GPs’ experiences of opioid prescribing for CNCP as an ongoing ethical conflict and dilemma where they have to navigate between patient autonomy, the risk of harming the patient and balance compassion with control. The second theme illustrates how GPs often feel isolated and unsupported yet indispensable. Overall, despite recognizing opioids as harmful, GPs continue prescribing to maintain therapeutic alliances and manage limitations in a fragmented health care organization where they perceive themselves as the last resort, obligated to alleviate their patients’ pain.

In line with previous studies [28,29,41–43], our findings indicate that the ongoing practice of prescribing compels GPs to endure an internal conflict between engaging in an action that may cause harm, i.e. LTOT, or adhering to their professional judgment that opioids should not be prescribed for CNCP. For instance, Toye et al. identified an overarching theme of ‘ambiguity’ in their qualitative evidence synthesis of health care providers’ experiences with prescribing opioids for CNCP. They emphasized the ongoing balancing act they must master in this context [29]. This ongoing balance act was described by the GPs in our study as arduous and challenging, creating a reluctance to prescribe opioids for CNCP. However, opioid tapering was uncommon and was described as too strenuous for both the patient and the GP to be feasible. Hence, an ethical dilemma had to be endured and was a source of moral distress among some of the GPs. Moral distress has been defined as ‘a phenomenon that occurs when *any* health care professional recognizes an ethical problem, feels the responsibility to respond to it, but cannot act or speak up in a way that is consistent with their professional integrity’ [55]. This evokes adverse emotional responses, including anger, frustration and a sense of powerlessness. Those feelings can explain the negative effects on GPs’ well-being when feeling forced to prescribe opioids as found in previous studies as well as in this study [42,43,56]. When left unresolved, moral distress can ‘provoke denial of the situation or one’s role in it’ [55]. The severity of this ethical tension becomes particularly apparent when the GPs in our study reflect on the act of renewing opioid prescriptions and the responsibility of harm. When prescriptions are renewed, the responsibility is shifted onto the patient or onto colleagues/the broader health care organization. The wish for support from authorities, including clinical guidelines, can be understood as strategies to safeguard against future ethical dilemmas related to prescription renewals.

Prescribing opioids for CNCP, opioid tapering and discontinuation of prescribing can all be understood as constant conflicts spanning intra- and interpersonal, as well as organizational, levels. This places sustained pressure on GPs’ ability to provide safe and effective care. This phenomenon has previously been studied by Johnsson et al. [57]. To understand how GPs make decisions – sometimes deviating from standard routines or guidelines – they propose that GPs navigate problematic situations by making three professional–moral judgments: ‘Shall I see what is before me *(the patient)*, or take a bird’s-eye view (the current issue is just one among many)? Shall I intervene, or stay my hand? And do I need to speak up, or should I rather shut up?’ According to our findings, the GPs choose to ‘take a bird’s eye view’ rather than see what is before them, which may explain asking for outside authorities/guidelines and the de-prioritization of both treatment evaluation and considering tapering.

The GPs primarily attribute the de-prioritization of these tasks to a lack of time. This appears to stem from an inherent expectation that the process will be too time-consuming and requires the physician to invest in a relationship founded on trust (*…and indispensable*), which may, however, ultimately lead to conflict *(The battlefield of opioid prescribing).* Prejudiced attitudes or preconceived assumptions about patients and consultations have been highlighted in earlier research, including that they contribute to stigma [58]. This has been found to be a fundamental cause of population health inequities in the health care context [58] and may promote the interpersonal conflicts. It also can explain the de-prioritization that our results demonstrate. Previous studies have highlighted the need for improved communication skills among GPs related to opioids and CNCP. A lack of trust has been shown to go both ways, from health care provider to patient and in reverse [29,59,60]. This is also described within *The battlefield of opioid prescribing*; where mutual trust is a critical factor which the GPs find hard to achieve when opioids are involved. When the GP decides to end opioid therapy, this may jeopardize the therapeutic relationship with the patient. Given that many GPs perceive themselves as the patient’s last resort, refraining from discontinuation may therefore appear to be a reasonable course of action. However, although establishing mutual trust was described as challenging, it was not regarded as unattainable.

The limited presence of discontinuation of opioid prescribing in the narratives may also be interpreted as a tendency among GPs to act in accordance with an idealized image of the physician – one who prioritizes close relationships and collaboration with the patient – rather than focusing on the prevention of potential opioid-related harms. This idealized self-image of being indispensable resonates with findings from international studies, including those reported by Keller et al. [35]. The GPs in their study ‘prided’ themselves on their relational and collaborative work with the patients to decrease doses of opioids that they found unsafe and managing psychosocial issues. This was in a health care system where other prescribers in the study were less willing to manage patients on LTOT. The development of professional identity is influenced by multiple factors [61]. Nevertheless, medical students are shaped into roles that are deeply embedded in societal power structures, illustrating that medical professionalization is not a neutral process [62]. Physicians are not only experts on the body but also bearers of social norms. These norms were evident in the themes *Legitimizing opioid prescribing practices* and *The last resort*, where GPs depicted themselves as physicians who believe that most pathological conditions can and should be treated pharmacologically. This perspective may help explain why GPs did not prioritize multi-disciplinary teams and collaboration, a finding that contrasts with previous studies conducted in contexts where alternatives to opioid treatment were scarce and actively sought [22,28, 63,64]. Instead, GPs in our study emphasized that both patients and other healthcare professionals depended on them, and ultimately, treatment decisions were regarded as the GP’s responsibility. This highlights how professional norms and organizational structures shape prescribing practices, underscoring the need to address attitudinal and systemic barriers when promoting multi-disciplinary collaboration in pain management.

Pharmaceuticals (and prescriptions) can be seen as the GP/physician’s unique prerogative when it comes to treatment (where other health professionals have other tools). This can be the reason that many conditions, including pain, are treated with pharmaceuticals in an attempt to ‘cure’. Thus, it is the role of the GP to treat pain pharmacologically, given that pain can be explained or the patient’s suffering is perceived as overwhelming. In the short term, this may appear to be a straightforward and convenient solution. However, in the long term, it risks imposing considerable adverse consequences on society, the healthcare system and individual patients.

The combination of feeling solely responsible for the patient’s overall care (*‘The last resort’*) and perceiving pharmacological treatment as their most effective available tool may contribute to the continued use of opioids in patients with CNCP. However, based on current evidence regarding effective treatments for CNCP, it may be argued that the GP’s role as indispensable – or, as previously described, as a ‘healer’ – is not entirely applicable in this context [64]. As argued by Bäckryd [65], the patient–physician relationship within the context of CNCP and opioid therapy would benefit from the GPs/physicians acting as a ‘Helping Professional’ rather than a ‘Professional helper’. This reflects the application of medical competence and an understanding that pain is a powerful driver of behavior. At the same time, it demonstrates the maintenance of professional integrity by communicating to the patient that chronic pain does not always have a straightforward solution, rather than yielding to the impulse that ‘something must be done’ [65]. Furthermore, in today’s society and healthcare, it is a much more up-to-date idea of the patient having shared responsibility for health, treatment and decisions.

## 6. Strengths and limitations

A strength of this study is the purposive sampling strategy which enabled the inclusion of participants with diversity in terms of working experience, type of employment (permanent, temporary), size and geographical location of health center (urban/rural). This diversity enriched the data and contributed to a broader understanding of the phenomenon under investigation. However, since the participants largely shared similar experiences, recruitment based on prescribing data could have provided additional insights into variations in clinical practice and decision-making. At the time of the interviews, AS had worked with or knew many of the participants who were recruited as GP colleagues. This may have increased participants’ willingness to take part in the study, despite a limited readiness to share their experiences in depth. On the other hand, it was perceived that participants were generally open and most were able to reflect on their own shortcomings, suggesting that they felt comfortable in the interview situation.

The interview guide was developed based on previous studies as well as the research team’s own experiences. Given the group’s broad clinical expertise in pain management, addiction and general practice, the involvement of the research team in designing the guide was considered a strength. Since AS had limited prior experience conducting interviews, this could be considered a limitation. On the other hand, AS’s familiarity with the issues at hand enabled her to guide the discussion in productive directions, which may also be considered a methodological strength. Conversely, given the sensitive topic of prescribing opioids for CNCP, it is possible that the informants sought to present themselves as competent and conscientious colleagues and did not disclose their most delicate personal limitations.

Given the researcher’s familiarity with the phenomenon and the context of the study, there was a risk that personal experiences could influence the interpretation and analysis of the data. To minimize this risk, MP and HL read all the interviews and SKS reviewed selected interviews and the interpretation of data was discussed in repeated meetings. It was considered a strength that the analysis team included members from different professional fields.

## 7. Conclusions

Opioid prescribing in CNCP is experienced as entailing ethical dilemmas and structural constraints. GPs described a constant balancing act between organizational demands and patient needs. This often includes negotiating principles of autonomy, beneficence, non-maleficence and justice when faced with requests to continue opioid therapy for CNCP. The GP-role was perceived as the ‘last resort’ within the healthcare system. This fostered a sense of sole responsibility for patient care and limited opportunities for delegation or collaborative approaches, and reinforced reliance on opioids as a pragmatic solution. Our findings underscore how underlying structures and professional norms shape prescribing practices and constrain multi-disciplinary collaboration. Addressing these attitudinal and systemic barriers is essential for developing sustainable strategies for chronic pain management in primary care.

## 8. Clinical implications and further research

To improve care for patients with CNCP and opioid therapy, fostering multi-disciplinary teamwork is essential. However, current organizational structures and prevailing GP attitudes present significant barriers that must be addressed. Future studies in Sweden should evaluate the impact of providing PCCs with support to build a multi-disciplinary team around patients with complex conditions, such as CNCP and opioid treatment, with the patient as an active partner. One example is an ongoing clinical study (Opi-Prim) that aims to optimize pain management in patients with LTOT for CNCP in primary care through a person-centered and team-based approach involving a GP, psychologist, physiotherapist, pharmacists and care manager [66].

## Appendix 1. Interview guide

1. What is your view on opioid prescribing for chronic pain in primary care?
2. How do you reason when faced with the decision to renew an opioid prescription for a patient with long-term non-cancer-­related pain?

*Which diagnoses and clinical indications are considered appropriate for initiating long-term opioid therapy? How do you approach diagnostic valuation in such cases? Which opioids? Special patient groups? Assessment of addiction potential?*

1. When do you reflect on reducing or discontinuing long-term opioid treatment? How do you approach this process?

*Which alternatives and tools do you use? Guidelines? How successful have previous attempts been? – What challenges have you encountered? – What would help you to discontinue opioid treatment? What is the most important need?*

1. How do you view collaboration with other professionals at the primary care center when it comes to managing such patients?

*To what extent do you usually seek support from other professionals? What motivates this? How could psychologists, social workers, nurses, physiotherapists or others contribute? Specifically, regarding clinical pharmacists, a relatively new profession in primary care* – *do you have experience collaborating with clinical pharmacists?*

1. What would you like to add that you feel is relevant and has not yet been discussed?

**Table 1.** Characteristics of participants, workplace and conditions for the interview (*n* = 12).

Characteristic	Value
Age, years (mean (range))	51 (33–67)
Gender, *n* (woman/man)	7/5
Work experience in primary care, years (mean (range))	13 (2–34)
Position, *n* (GP/GP resident)	11/1
Employment type, *n* (permanent/temporary)	10/2
Health center ownership, *n* (public/private)	7/5
Location of health center, *n* (urban/rural)^a^	6/6
Interview condition, *n* (face-to-face/digitally/by phone)	8/3/1

^a^ ’Urban’ denotes the urban area (population approximately 175,000), whereas ‘Rural’ denotes adjacent municipalities with populations ranging from approximately 15,000 to 50,000.

## References

[1] Definitions of chronic pain syndromes. International Association for the Study of Pain (IASP); 2026 [Internet] [cited 2026 Feb 18]. Available from: https://www.iasp-pain.org/advocacy/definitions-of-chronic-pain-syndromes/

[2] Breivik H, Collett B, Ventafridda V, et al. Survey of chronic pain in Europe: prevalence, impact on daily life, and treatment. Eur J Pain. 2006;10(4):287–333. doi: 10.1016/j.ejpain.2005.06.009. 16095934

[3] Global burden of 369 diseases and injuries in 204 countries and territories, 1990–2019: a systematic analysis for the Global Burden of Disease Study 2019. Lancet. 2020;396(10258):1204–1222. doi: 10.1016/S0140-6736(20)30925-9. 33069326 PMC7567026

[4] Cohen SP, Vase L, Hooten WM. Chronic pain: an update on burden, best practices, and new advances. Lancet. 2021;397(10289):2082–2097. doi: 10.1016/S0140-6736(21)00393-7. 34062143

[5] Läkemedelsverket. Läkemedelsbehandling av långvarig smärta hos vuxna – behandlingsrekommendation. Uppsala: Läkemedelsverket; 2017.

[6] Dowell D, Ragan KR, Jones CM, et al. CDC clinical practice guideline for prescribing opioids for pain – United States, 2022. MMWR Recomm Rep. 2022;71(3):1–95. doi: 10.15585/mmwr.rr7103a1. PMC9639433 36327391

[7] Bäckryd E. Att navigera mellan opioidrädsla och opiocentrism i dagens vård. Läkartidningen; 2022 [Internet] [cited 2023 Oct 2]. Available from: https://lakartidningen.se/klinik-och-vetenskap-1/artiklar-1/temaartikel/2022/01/att-navigera-mellan-opioidradsla-och-opiocentrism-i-dagens-vard/35491616

[8] Busse JW, Wang L, Kamaleldin M, et al. Opioids for chronic noncancer pain. JAMA. 2018;320(23):2448–2460. doi: 10.1001/jama.2018.18472. 30561481 PMC6583638

[9] Krebs EE, Gravely A, Nugent S, et al. Effect of opioid vs nonopioid medications on pain-related function in patients with chronic back pain or hip or knee osteoarthritis pain. JAMA. 2018;319(9):872–882. doi: 10.1001/jama.2018.0899. 29509867 PMC5885909

[10] Manchikanti L, Kaye AM, Knezevic NN, et al. Comprehensive, evidence-based, consensus guidelines for prescription of opioids for chronic non-cancer pain from the American Society of Interventional Pain Physicians (ASIPP). Pain Physician. 2023;26(7S):S7–S126. 38117465

[11] Veiga DR, Monteiro-Soares M, Mendonça L, et al. Effectiveness of opioids for chronic noncancer pain: a two-year multicenter, prospective cohort study with propensity score matching. J Pain. 2019;20(6):706–715. doi: 10.1016/j.jpain.2018.12.007. 30597203

[12] Altawili AA, Altawili MA, Alzarar AH, et al. Adverse events of the long-term use of opioids for chronic non-cancer pain: a narrative review. Cureus. 2024;16(1):e51475. doi: 10.7759/cureus.51475. 38298287 PMC10830133

[13] Chou R, Turner JA, Devine EB, et al. The effectiveness and risks of long-term opioid therapy for chronic pain: a systematic review for a National Institutes of Health Pathways to Prevention Workshop. Ann Intern Med. 2015;162(4):276–286. doi: 10.7326/M14-2559. 25581257

[14] Förskrivning av opioider i Sverige Användning över tid. Rapp Från Läkemedelsverket. Dnr: 4.3.1-2018-102265; 2020 [Internet]. Available from: https://www.lakemedelsverket.se/4abcb6/globalassets/dokument/publikationer/lakemedelsprodukter-och-narkotika/rapport-forskrivning-av-opioider-i-sverige-anvandning-over-tid.pdf

[15] Balikci Z, Kondratova U, Picco L, et al. Opioid prescribing prevalence and initiation rates in Victoria, Australia: insights from primary care data during a period of opioid policy changes (2017–2022). Int J Clin Pharm. 2025;47(2):443–451. doi: 10.1007/s11096-024-01849-0. 39718760

[16] Trecki J. A perspective regarding the current state of the opioid epidemic. JAMA Netw Open. 2019;2(1):e187104. doi: 10.1001/jamanetworkopen.2018.7104. 30657528

[17] Morone NE, Weiner DK. Pain as the 5th vital sign: exposing the vital need for pain education. Clin Ther. 2013;35(11):1728–1732. doi: 10.1016/j.clinthera.2013.10.001. 24145043 PMC3888154

[18] Förskrivning av opioider i Sverige Läkemedel, doser och diagnoser.Uppsala: Läkemedelsverket; 2020 (Dnr: 4.3.1-2018-102265).

[19] Bäckryd E. Dynamiken i förskrivningen av opioider i Sverige 2000–2015. Stockholm: Läkartidningen 2017114EFUE; 2017. 28485763

[20] Rolová G, Skurtveit S, Handal M, et al. Trends in opioid prescribing in Scandinavian countries from 2010 to 2023: insights from multi-metric evaluation. Br J Clin Pharmacol. 2025;91(12):3341–3352. doi: 10.1002/bcp.70177. 40702924 PMC12648362

[21] Fugelstad A, Ågren G, Ramstedt M, et al. Oxycodone-related deaths in Sweden 2006–2018. Drug Alcohol Depend. 2022;234:109402. doi: 10.1016/j.drugalcdep.2022.109402. 35306392

[22] de Kleijn L, Jansen-Groot Koerkamp EAW, van der Kooij I, et al. Exploring the facilitators and barriers in opioid deprescribing for non-cancer pain treatment experienced by general practitioners: a qualitative study. Eur J Pain. 2024;28(7):1101–1115. doi: 10.1002/ejp.2243. 38287911

[23] Mathieson S, Maher CG, Ferreira GE, et al. Deprescribing opioids in chronic non-cancer pain: systematic review of randomised trials. Drugs. 2020;80(15):1563–1576. doi: 10.1007/s40265-020-01368-y. 32737739

[24] Henrik G, Patrik M, Anders H, et al. Tapering of prescribed opioids in patients with long-term non-malignant pain (TOPIO)—efficacy and effects on pain, pain cognitions, and quality of life: a study protocol for a randomized controlled clinical trial with a 12-month follow-up. Trials. 2021;22(1):503. doi: 10.1186/s13063-021-05449-5. 34321058 PMC8318331

[25] Agnoli A, Xing G, Tancredi DJ, et al. Association of dose tapering with overdose or mental health crisis among patients prescribed long-term opioids. JAMA. 2021;326(5):411–419. doi: 10.1001/jama.2021.11013. 34342618 PMC8335575

[26] Farrar JT, Bilker WB, Cochetti PT, et al. Evaluating the stability of opioid efficacy over 12 months in patients with chronic noncancer pain who initially demonstrate benefit from extended release oxycodone or hydrocodone: harmonization of Food and Drug Administration patient-level drug safety study data. Pain. 2022;163(1):47–57. doi: 10.1097/j.pain.0000000000002331. 34261978 PMC8675053

[27] Conroy A, Bilker WB, DeLorey I, et al. Predictors of response to full agonist opioids in enriched enrollment randomized withdrawal clinical trials: a participant-level data meta-analysis. Pain. 2022;167(2):320–325. doi: 10.1097/j.pain.0000000000003803. 40893014

[28] Punwasi R, de Kleijn L, Rijkels-Otters JBM, et al. General practitioners’ attitudes towards opioids for non-cancer pain: a qualitative systematic review. BMJ Open. 2022;12(2):e054945. doi: 10.1136/bmjopen-2021-054945. PMC8808445 35105588

[29] Toye F, Seers K, Tierney S, et al. A qualitative evidence synthesis to explore healthcare professionals’ experience of prescribing opioids to adults with chronic non-malignant pain. BMC Fam Pract. 2017;18(1):94. doi: 10.1186/s12875-017-0663-8. 29178843 PMC5702226

[30] Cai Q, Grigoroglou C, Allen T, et al. Interventions to reduce opioid use for patients with chronic non-cancer pain in primary care settings: a systematic review and meta-analysis. PLOS One. 2024;19(10):e0300954. doi: 10.1371/journal.pone.0300954. 39423192 PMC11488744

[31] Avery N, McNeilage AG, Stanaway F, et al. Efficacy of interventions to reduce long term opioid treatment for chronic non-cancer pain: systematic review and meta-analysis. BMJ. 2022;377:e066375. doi: 10.1136/bmj-2021-066375. 35379650 PMC8977989

[32] de Kleijn L, Pedersen JR, Rijkels-Otters H, et al. Opioid reduction for patients with chronic pain in primary care: systematic review. Br J Gen Pract. 2022;72(717):e293–e300. doi: 10.3399/BJGP.2021.0537. 35023850 PMC8843401

[33] Bathia NS, McAskill RE, Hancox JE, et al. Collaboration between adult patients and practitioners when making decisions about prescribing opioid medicines for chronic non-cancer pain in primary care: a scoping review. Br J Pain. 2022;16(1):119–126. doi: 10.1177/20494637211025560. 35111320 PMC8801689

[34] Mackey K, Anderson J, Bourne D, et al. Benefits and harms of long-term opioid dose reduction or discontinuation in patients with chronic pain: a rapid review. J Gen Intern Med. 2020;35(Suppl. 3):935–944. doi: 10.1007/s11606-020-06253-8. PMC7728933 33145689

[35] Keller MS, Jusufagic A, Nuckols TK, et al. Understanding clinicians’ decisions to assume prescriptions for inherited patients on long-term opioid therapy: a qualitative study. Pain Med. 2020;21(11):3187–3198. doi: 10.1093/pm/pnaa045. 32186728 PMC8453626

[36] Personcentrerat och sammanhållet vårdförlopp Smärta – långvarig, hos vuxna. Stockholm: Sveriges Regioner i Samverkan; 2022.

[37] Wändell P, Carlsson AC, Wettermark B, et al. Most common diseases diagnosed in primary care in Stockholm, Sweden, in 2011. Fam Pract. 2013;30(5):506–513. doi: 10.1093/fampra/cmt033. 23825186

[38] Finley CR, Chan DS, Garrison S, et al. What are the most common conditions in primary care? Can Fam Physician. 2018;64(11):832–840. 30429181 PMC6234945

[39] Sweden: health system summary 2024. OBS. European Observatory on Health Systems and Policies; 2025 [Internet] [cited 2025 Nov 10]. Available from: https://eurohealthobservatory.who.int/publications/i/sweden-health-system-summary-2024

[40] Rehabilitation guarantee: evaluation of the initiation of the national rehabilitation guarantee for common mental disorders and backpain. Karolinska Institutet; 2025 [Internet] [cited 2025 Nov 10]. Available from: https://ki.se/en/imm/research/units-at-imm/unit-of-intervention-and-implementation-research-in-occupational-health/rehabilitation-guarantee-evaluation-of-the-initiation-of-the-national-rehabilitation-guarantee-for-common-mental-disorders-and-backpain

[41] Ljungvall H, Öster C, Katila L, et al. “Opioids are opioids” – a phenomenographic analyses of physicians’ understanding of what makes the initial prescription of opioids become long-term opioid therapy. Scand J Pain. 2022;22(3):494–505. doi: 10.1515/sjpain-2021-0171. 35172418

[42] Ekelin E, Hansson A. The dilemma of repeat weak opioid prescriptions – experiences from Swedish GPs. Scand J Prim Health Care. 2018;36(2):180–188. doi: 10.1080/02813432.2018.1459241. 29693484 PMC6066274

[43] Bendtsen P, Hensing G, Ebeling C, et al. What are the qualities of dilemmas experienced when prescribing opioids in general practice? Pain. 1999;82(1):89–96. doi: 10.1016/S0304-3959(99)00036-6. 10422664

[44] Tong A, Sainsbury P, Craig J. Consolidated criteria for reporting qualitative research (COREQ): a 32-item checklist for interviews and focus groups. Int J Qual Health Care. 2007;19(6):349–357. doi: 10.1093/intqhc/mzm042. 17872937

[45] Silverman D. Interpreting qualitative data. 6th ed. London: SAGE Publications; 2020.

[46] Braun V, Clarke V. Reflecting on reflexive thematic analysis. Qual Res Sport Exerc Health. 2019;11(4):589–597. doi: 10.1080/2159676X.2019.1628806.

[47] Beauchamp TL, Childress JF. Principles of biomedical ethics. 8th ed. New York: Oxford University Press; 2019.

[48] Plan ska tas fram: tak för antal listade patienter per läkare; 2025 [Internet] [cited 2025 Nov 28]. Available from: https://regionuppsala.se/politik-och-paverkan/pressrum/2022/juni/plan-ska-tas-fram-tak-for-antal-listade-patienter-per-lakare/

[49] Braun V, Clarke V. To saturate or not to saturate? Questioning data saturation as a useful concept for thematic analysis and sample-size rationales. Qual Res Sport Exerc Health. 2021;13(2):201–216. doi: 10.1080/2159676X.2019.1704846.

[50] Braun V, Clarke V. Can I use TA? Should I use TA? Should I not use TA? Comparing reflexive thematic analysis and other pattern-based qualitative analytic approaches. Couns Psychother Res. 2021;21(1):37–47. doi: 10.1002/capr.12360.

[51] Braun V, Clarke V. Using thematic analysis in psychology. Qual Res Psychol. 2006;3(2):77–101. doi: 10.1191/1478088706qp063oa.

[52] Etikprovningsmyndigheten; 2023. Available from: https://etikprovningsmyndigheten.se/wp-content/uploads/2024/05/Guide-to-the-ethical-review_webb.pdf

[53] Regulation – 2016/679 – EN – gdpr – EUR-Lex; 2025 [Internet] [cited 2025 Feb 20]. Available from: https://eur-lex.europa.eu/eli/reg/2016/679/oj/eng

[54] Noble H, Smith J. Issues of validity and reliability in qualitative research. Evid Based Nurs. 2015;18(2):34–35. doi: 10.1136/eb-2015-102054. 25653237

[55] Carse A, Thomas TA, Rushton CH. What is moral distress? Understanding context, sources, and consequences. In: Rushton CH, editor. Moral resilience: transforming moral suffering in healthcare. Oxford: Oxford University Press. p. 29–61; 2024.

[56] Prathivadi P, Barton C, Mazza D. The opioid-prescribing practices of Australian general practice registrars: an interview study. Fam Pract. 2021;38(4):473–478. doi: 10.1093/fampra/cmaa148. 33506867

[57] Johnsson L, Höglund AT, Nordgren L. The voice of the profession: how the ethical demand is professionally refracted in the work of general practitioners. BMC Med Ethics. 2023;24(1):75. doi: 10.1186/s12910-023-00958-1. 37752505 PMC10523728

[58] McCradden MD, Vasileva D, Orchanian-Cheff A, et al. Ambiguous identities of drugs and people: a scoping review of opioid-related stigma. Int J Drug Policy. 2019;74:205–215. doi: 10.1016/j.drugpo.2019.10.005. 31671303

[59] Buchman DZ, Ho A, Illes J. You present like a drug addict: patient and clinician perspectives on trust and trustworthiness in chronic pain management. Pain Med. 2016;17(8):1394–1406. doi: 10.1093/pm/pnv083. 26759389 PMC4975016

[60] Buchman DZ, Ho A. What’s trust got to do with it? Revisiting opioid contracts. J Med Ethics. 2014;40(10):673–677. doi: 10.1136/medethics-2013-101320. 24014642

[61] Reissner S, Armitage-Chan E. Manifestations of professional identity work: an integrative review of research in professional identity formation. Stud High Educ. 2024;49(12):2707–2722. doi: 10.1080/03075079.2024.2322093.

[62] Smith-Oka V. Becoming gods: medical training in Mexican hospitals. New Brunswick: Rutgers University Press; 2021 [Internet] [cited 2024 Jun 12]. Available from: http://ebookcentral.proquest.com/lib/uu/detail.action?docID=6661533

[63] Prathivadi P, Barton C, Mazza D. Qualitative insights into the opioid prescribing practices of Australian GP. Fam Pract. 2020;37(3):412–417. doi: 10.1093/fampra/cmz083. 31768532

[64] Desveaux L, Saragosa M, Kithulegoda N, et al. Family physician perceptions of their role in managing the opioid crisis. Ann Fam Med. 2019;17(4):345–351. doi: 10.1370/afm.2413. 31285212 PMC6827657

[65] Bäckryd E. “Professional helper” or “helping professional?” The patient–physician relationship in the chronic pain setting, with special reference to the current opioid debate. J Contin Educ Health Prof. 2016;36(2):133–137. doi: 10.1097/CEH.0000000000000062. 27116642

[66] Kempen T. Improved treatment for patients with long-term opioid therapy for non-cancer pain in primary care (Opi-Prim) [Clinical trial registration]. clinicaltrials.gov; 2025 [Internet] [cited 2025 Dec 5]. Report No. NCT06856733. Available from: https://clinicaltrials.gov/study/NCT06856733

